# Chaperone function in antigen presentation by MHC class I molecules—tapasin in the PLC and TAPBPR beyond

**DOI:** 10.3389/fimmu.2023.1179846

**Published:** 2023-06-15

**Authors:** David H. Margulies, Jiansheng Jiang, Javeed Ahmad, Lisa F. Boyd, Kannan Natarajan

**Affiliations:** Molecular Biology Section, Laboratory of Immune System Biology, National Institute of Allergy and Infectious Diseases, National Institutes of Health, Bethesda, MD, United States

**Keywords:** antigen presentation, tapasin, TAPBPR, MHC class I, peptide loading complex, structural immunology

## Abstract

Peptide loading of MHC-I molecules plays a critical role in the T cell response to infections and tumors as well as to interactions with inhibitory receptors on natural killer (NK) cells. To facilitate and optimize peptide acquisition, vertebrates have evolved specialized chaperones to stabilize MHC-I molecules during their biosynthesis and to catalyze peptide exchange favoring high affinity or optimal peptides to permit transport to the cell surface where stable peptide/MHC-I (pMHC-I) complexes are displayed and are available for interaction with T cell receptors and any of a host of inhibitory and activating receptors. Although components of the endoplasmic reticulum (ER) resident peptide loading complex (PLC) were identified some 30 years ago, the detailed biophysical parameters that govern peptide selection, binding, and surface display have recently been understood better with advances in structural methods including X-ray crystallography, cryogenic electron microscopy (cryo-EM), and computational modeling. These approaches have provided refined mechanistic illustration of the molecular events involved in the folding of the MHC-I heavy chain, its coordinate glycosylation, assembly with its light chain, β_2_-microglobulin (β_2_m), its association with the PLC, and its binding of peptides. Our current view of this important cellular process as it relates to antigen presentation to CD8^+^ T cells is based on many different approaches: biochemical, genetic, structural, computational, cell biological, and immunological. In this review, taking advantage of recent X-ray and cryo-EM structural evidence and molecular dynamics simulations, examined in the context of past experiments, we attempt a dispassionate evaluation of the details of peptide loading in the MHC-I pathway. By critical evaluation of several decades of investigation, we outline aspects of the peptide loading process that are well-understood and indicate those that demand further detailed investigation. Further studies should contribute not only to basic understanding, but also to applications for immunization and therapy of tumors and infections.

## 1 Introduction

**Figure 1:**
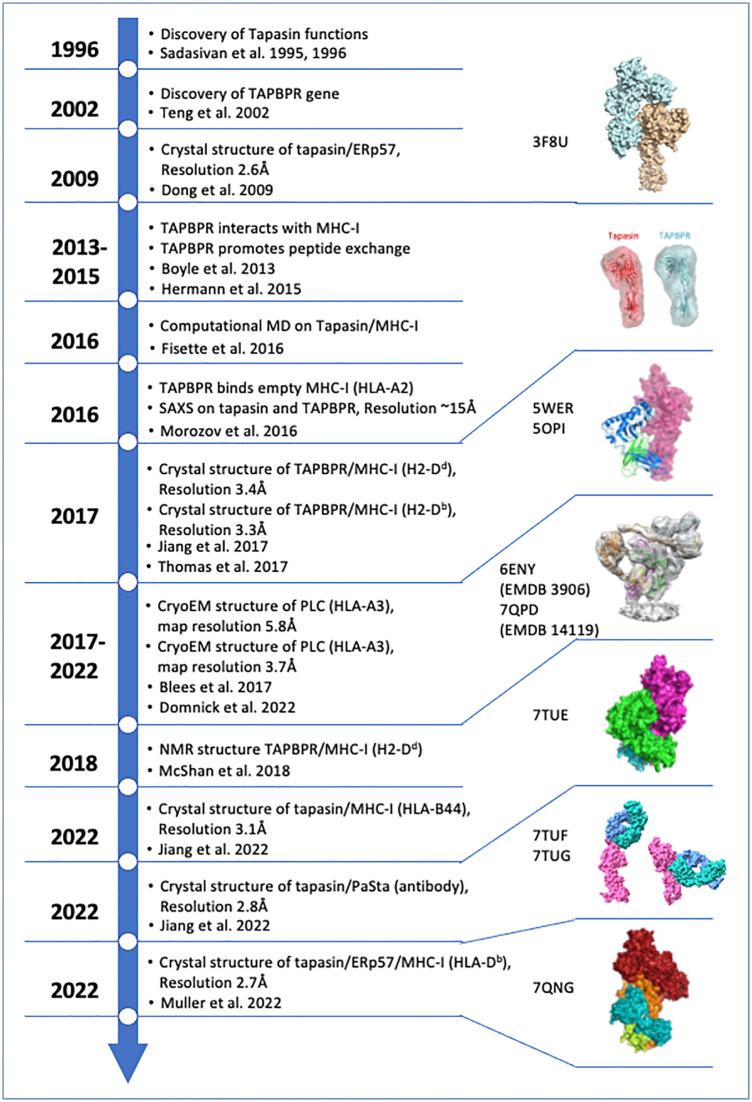
Timeline of structures of key functional and structural findings concerning Tapasin and TAPBPR chaperones. Year of publication of relevant indicated papers is shown and annotated. Summary of key structures and conclusions are shown. Relevant PDB ID codes and EMDB numbers are indicated. The citations in the figure are referenced here as (8–22). Structural models referred to in this paper have been published and have been deposited in either the protein data bank (PBD) (rcsb.org (23)) or the electron microscopy data bank (EMDB) (24). All images were generated from PDB coordinates or EMDB maps using ChimeraX (25).

Survival amidst a host of infectious agents and the scourge of neoplasia requires multicellular organisms to recognize pathogens, pathogen-infected cells, and dysregulated cancer cells. The vertebrate immune system has evolved a complex and effective molecular and cellular system to accomplish this formidable task, and one group of effective solutions includes the means to discriminate cells that no longer function normally. Central to this recognition function are cell surface molecules encoded by the major histocompatibility complexes of higher vertebrates, known collectively as MHC molecules and functionally discerned as two heterodimeric classes, MHC-I and MHC-II. Classical MHC-I molecules, designated H2-K, -D, and -L in the mouse and HLA-A,-B, and -C in the human are expressed on the surface of virtually all nucleated cells, paired with a monomorphic chain, β_2_m (1). The MHC-I heavy chains, ~ 46 kD in size, are most remarkable for their high degree of polymorphism (some 25,000 HLA class I and 10,000 HLA class II alleles are now recognized by the Immuno Polymorphism Database (IPD) (2, 3)). Our focus for this review is the MHC-I molecules and the mechanism by which they are loaded with antigenic peptides. The collective process of generating, binding, exchanging, and displaying peptide/MHC (pMHC) complexes at the cell surface is designated “antigen processing and presentation,” which has been reviewed extensively elsewhere (4–7). The goal of this review is to offer perspective on recent experiments that address mechanistic details of peptide loading onto MHC-I. **Figure 1** offers a timeline of some of the major experimental/structural insights concerning MHC-I peptide loading.

Our current understanding draws on extensive biochemical, genetic, and functional studies and focuses specifically on structural visualization from X-ray (8–11), small angle X-ray scattering (SAXS) (12) and cryo-EM (13, 14) methods. In addition, it draws on dynamic observations based on molecular dynamics simulations and nuclear magnetic resonance (NMR) spectroscopy (15, 26–28). We emphasize that the important functional unit in MHC-I peptide loading is the PLC, governed largely by the tapasin chaperone/editor. (Tapasin, also known as TAP binding protein (TAPBP), was originally identified in immunoprecipitation experiments showing that it bridged the transporter associated with antigen processing (TAP) to MHC-I (16)). Major insight into tapasin function has been gained more recently by studies of a similar molecule, TAP binding protein, related (TAPBPR), in large part because it has been more amenable to experimental study and manipulation.

The PLC consists of the heterodimer peptide transporter TAP1/2, the protein disulfide isomerase endoplasmic reticulum (ER) protein 57 (ERp57) (also known as PDIA3), the lectin chaperone calreticulin, and tapasin (16, 17). The TAP1/2 heterodimer is an ATP-dependent peptide transporter that shuttles peptides of length 8 to 16 (even as large as 40) derived from cytoplasmic degradation of proteins by the proteasome into the ER (29). TAP mutant mice are defective in MHC-I cell surface expression and antigen presentation, resulting in defective CD8^+^ T cell development (30). Following transport by TAP, peptides are subject to amino-terminal trimming by ER aminopeptidase (ERAP) 1 (31) or ERAP 2 (32) in the human, or ER associated with antigen processing (ERAAP) in the mouse (33). Cells or animals defective in the genes encoding tapasin (34–36) revealed a critical role for tapasin both in stabilizing unstable MHC-I molecules bound to low affinity peptides and in catalyzing peptide exchange in a process that selects for high affinity MHC-I peptides. Early biochemical and functional studies of tapasin and its covalent disulfide-mediated interaction with ERp57 (37) were substantiated by the determination of the three-dimensional structure of a molecular heterodimer of tapasin with ERp57 (18). This not only established the structural basis of tapasin and the tapasin/ERp57 association with PLC function but defined some of the functional regions of tapasin by careful mutagenic studies. Concurrent molecular modeling and dynamics studies pointed to the concerted roles of tapasin, ERp57, and calreticulin in stabilizing MHC-I and facilitating peptide loading (27, 38, 39). However, because of the lack of direct knowledge of the contacts of tapasin with the MHC molecule, a more complete understanding of the mechanisms involved in MHC-I chaperoning and catalysis of peptide exchange remained lacking.

Because a number of tapasin expression systems, though useful for examining tapasin interactions *in vitro* (40–43), proved unable to provide material suitable for high resolution structural studies of the interaction of tapasin with MHC-I, interest in tapasin and the PLC diverted to studies of the tapasin homologue, TAPBPR. The TAPBPR gene (on human chromosome 12p13.3) had been identified in a genetic region paralogous to the human MHC (at 6p21.3), and its encoded protein was some 20% identical to that of tapasin (19). Because of the similarity of predicted structural domains and potential for equivalence in function, several laboratories pursued biochemical and functional (12, 20), and eventually structural studies of TAPBPR (8, 10, 21, 44). These findings initiated renewed interest in the intricacies of MHC-I/chaperone interactions. Biochemical studies of TAPBPR established its catalytic role in peptide exchange, confirming the view that its mechanism would be similar to that of tapasin (12, 22).

More recently several major advances have orchestrated a return to understanding the details of tapasin/MHC-I interactions. These include the crystal structure determination of tapasin/MHC-I/ERp57 complex (11), the crystal structure of a tapasin/MHC-I complex (9), and the cryo-EM visualization of complete PLC preparations (13, 14). This review will aim to summarize some new findings based on studies with TAPBPR and unite their interpretation with structural studies of tapasin complexed with MHC-I as probed crystallographically, by cryo-EM, and computationally. Our approach will be primarily structural, focusing on the regions of TAPBPR and tapasin that have been identified to interact with MHC-I and other PLC components.

## 2 Tapasin and TAPBPR in chaperoning and peptide loading

### 2.1 Tapasin-identification of functional domains by mutagenesis and X-ray crystallography

**Figure 2:**
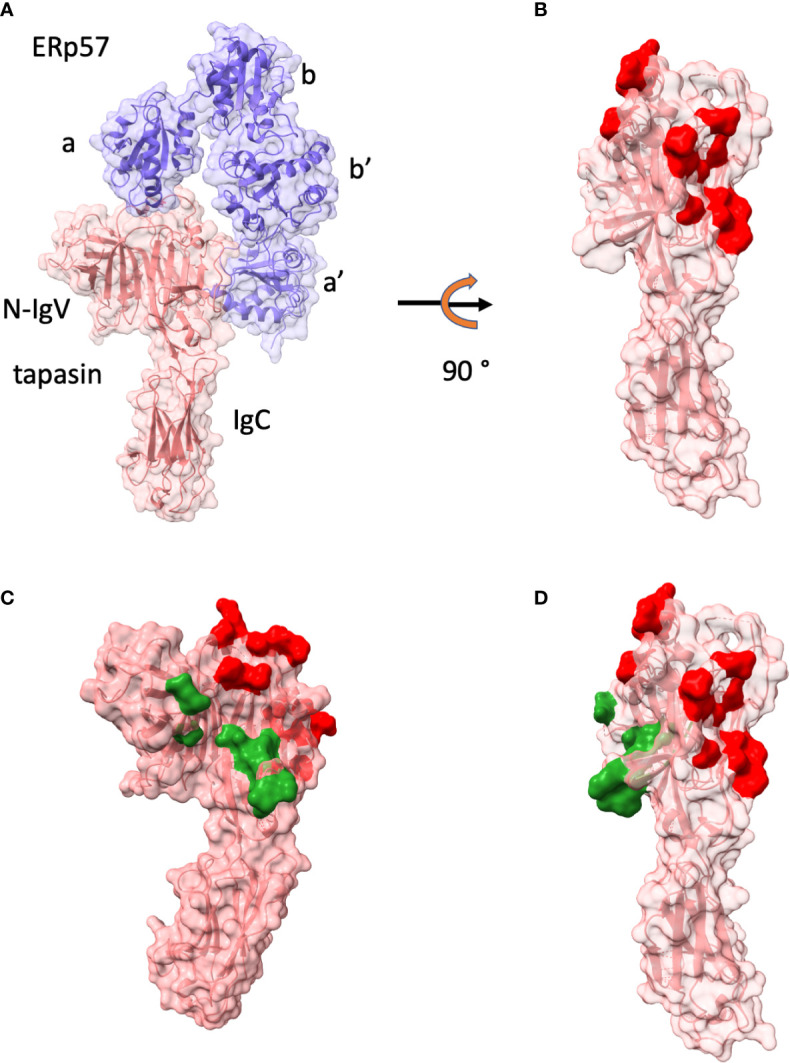
Tapasin structure and interface with ERp57 are shown. **(A)** Structure as ribbon and surface of tapasin (tan)/ERp57(purple) (PDBID: 3F8U (18)) and domains of ERp57 (a, b, b’, c) and of tapasin N-IgV and IgD are labelled. **(B)** tapasin only is displayed after 90° rotation. Red surface indicates residues of contact with ERp57. **(C)** tapasin surface is colored red for ERp57 contacts, and dark green for those identified by mutagenesis as reducing MHC-I surface expression in tapasin negative cell line (18). **(D)** illustration of tapasin in **(C)** rotated 90°.

As noted above, tapasin was first identified in studies of the TAP transporter as a molecule bridging MHC-I to TAP (16, 17, 45–47). X-ray crystal structure determination of tapasin bound to the oxidoreductase ERp57 revolutionized our understanding of the functional domains of tapasin (18). As shown in **Figure 2**, the 2.60 Å crystal structure of this complex revealed the surface of tapasin that interacts with ERp57, and, on the opposite face of the molecule, a large region available for interaction with MHC-I. The contacts of tapasin to ERp57 emphasized the stabilizing influence of ERp57 for tapasin. Further mutational analysis in this paper identified several regions of tapasin that affected complementation of cell surface expression of MHC-I (HLA-A, -B, and -C) in tapasin deficient cells (**Figures 2B, C**).

### 2.2 TAPBPR-a welcome and important diversion

Although tapasin function was well-studied with respect to MHC-I surface expression and peptide presentation, efforts to explore the structural mechanistic basis of peptide editing and MHC-I stabilization by tapasin languished until Boyle and colleagues diverted attention of the antigen presentation community to TAPBPR (20). Because of its higher affinity for MHC-I and somewhat better behavior as a recombinant molecule, TAPBPR proved to be amenable for biochemical/binding studies as well as small angle X-ray scattering (SAXS) studies (12, 22, 48). TAPBPR was shown to function as a catalyst in peptide exchange (12, 22) and to interact with MHC-I and UDP-glucose:glycoprotein glucosyltransferase 1 (UGT1) in a pathway that monitors the quality of MHC-I peptide loading (49). The SAXS studies confirmed the impression that tapasin and TAPBPR possess similar dimensions at low resolution, and binding studies indicated that TAPBPR had higher affinity for MHC-I molecules emptied of peptide by photolysis of photolabile peptides than for those bound to peptides. As predicted, TAPBPR revealed a hierarchy of exchangeability dependent on the intrinsic affinity of the MHC-I bound peptides. Additional studies revealed differences in the ability of distinct MHC-I allelomorphs to interact with and be catalyzed by TAPBPR (12, 50). Dynamic studies of the exchange process using NMR revealed a negative allosteric function of TAPBPR (51). A practical outcome of the recognition of TAPBPR as a peptide editor has been its technological use in mediating peptide exchange, either with recombinant molecules (52) or at the cell surface (53).

**Figure 3:**
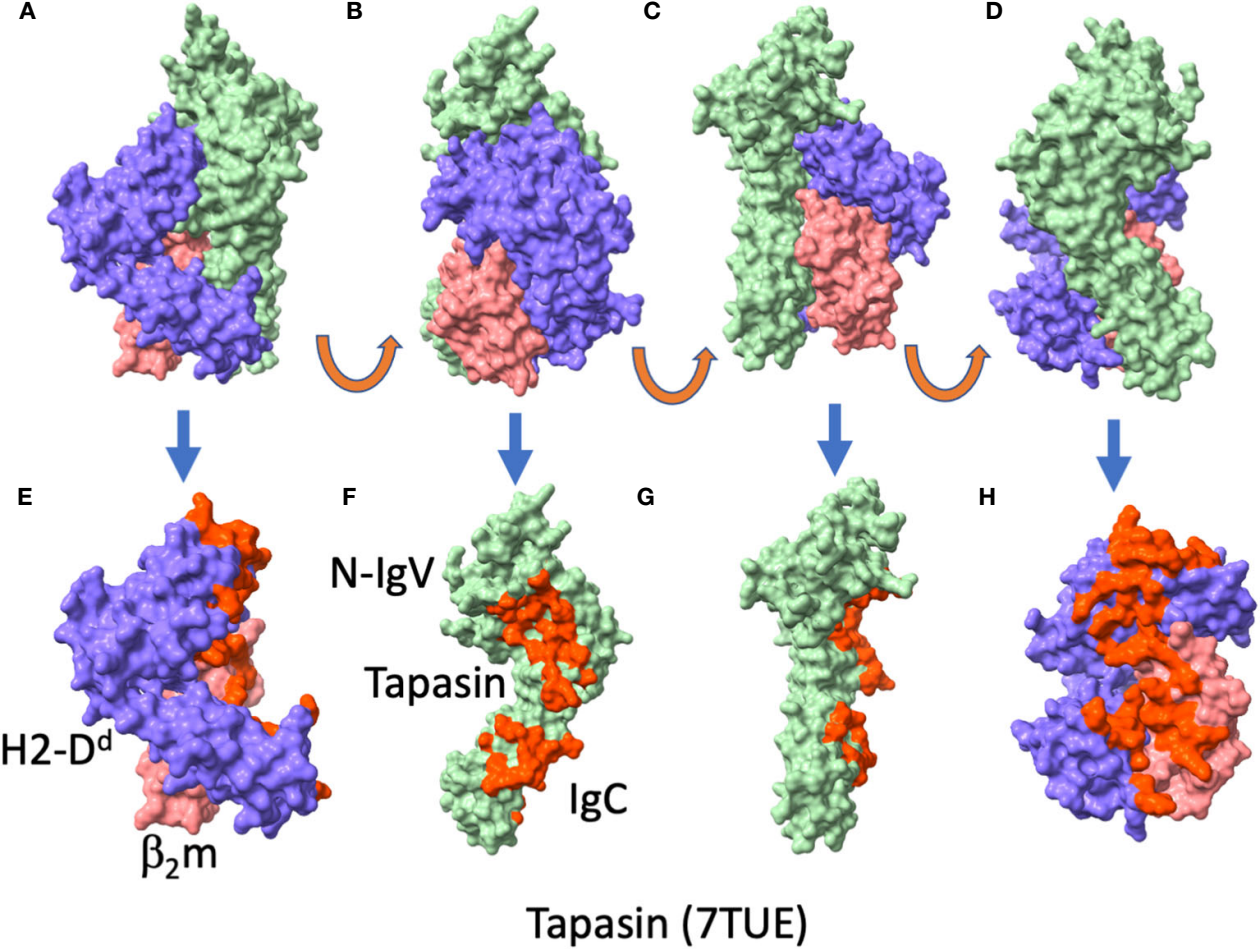
TAPBPR complexed with H2-D^d^ reveals broad interface. **(A-D)** surface rendition of H2-D^d^/β_2_m/TAPBPR complex (PDBID: 5WER (8), first complex in the asymmetric unit), H2-D^d^ (purple), β_2_m (coral), TAPBPR (light green), with successive panel showing 90° rotation. **(E)** complex with TAPBPR deleted. Residues of H2-D^d^/β_2_m that contact TAPBPR are colored red. **(F, G)** TAPBPR alone, with residues that contact H2-D^d^/β_2_m shown in red. **(F, G)** are related by 90° rotation. **(H)** H2- D^d^/β_2_m, with residues that contact TAPBPR colored red.

The structural studies noted above generated new paradigms for understanding chaperone function in peptide loading. The X-ray structures, one of a complex of human TAPBPR with the murine H2-D^b^ molecule (10), the other of a complex of human TAPBPR with mouse H2-D^d^ (8) were remarkably similar. Comparison of the TAPBPR component of the two structures, determined independently in the two laboratories, revealed great similarity as indicated by a root-mean-square-deviation (RMSD) of superposed backbone carbon atoms of 0.935 Å. **Figure 3** shows the TAPBPR/H2-D^d^ structure, showing how TAPBPR makes broad contact with the MHC-I molecule and its β_2_m light chain (**Figures 3E, H**). Contacts of MHC-I H and L chains to TAPBPR are also illustrated (**Figures 3F, G**). These X-ray structures were obtained by generating complexes between the TAPBPR and MHC-I molecules that had been either completely emptied by photolysis of a photolabile peptide (10), or by engineering a disulfide linkage between the α1 helix of the MHC-I with a C-terminal cysteine substitution in the truncated 5-mer peptide (8). Structures determined in the two laboratories revealed no consistent electron density in the binding groove, distortion of the empty binding groove by changes in the position of the α2-1 helix by at least 3 Å, and poor or non-existent electron density for a loop extending from residue 22 to 36 of TAPBPR. This loop, designated the scoop loop by some (10) clearly does not interact with the floor of the peptide binding groove at or near the F pocket which normally anchors the side chain of the C-terminal amino acid of the bound peptide. Some investigators have invoked a competitive function for this loop in TAPBPR (54), but others have argued that little evidence exists to substantiate such a model (51, 55). Indeed, NMR studies are consistent with the loop serving as a “peptide trap” by positioning itself above the MHC-I α1 and α2 helices (51). Another TAPBPR loop, extending from residues 209 to 213, interacts beneath the floor of the MHC-I peptide binding groove and plays a dynamic role in stabilizing peptide-free MHC-I molecules. In the structures determined by both laboratories, the disposition of a conserved tyrosine at position 84 of the MHC-I chain appears to play a role in either stabilizing bound peptide or in interacting with TAPBPR. The consistent feature of the TAPBPR structures was that they revealed extensive interactions of the membrane proximal IgC domain of TAPBPR with the membrane proximal Ig domains (α3 and β_2_m) of the MHC-I. Overall, X-ray crystallography studies of TAPBPR, complemented by detailed NMR studies (21, 51, 56) and computational studies (57, 58) have contributed to a clearer picture of some of the details of the mechanism of peptide loading. Despite considerable interest and the valuable lessons of MHC-I chaperones learned from the study of TAPBPR, the precise function of TAPBPR remains somewhat of an enigma, since cells deficient in its expression reveal little effect on MHC-I surface expression.

### 2.3 Return to tapasin, the major MHC-I chaperone

The first cryo-EM structures of the complete PLC, though of relatively low resolution (13), revealed the stoichiometry of the PLC to be consistent with that determined years earlier by antibody pull-down experiments that indicated a tapasin/MHC-I stoichiometry of either 2:1 or 2:2 in the PLC (59). These studies then set the stage for subsequent X-ray studies revealing the interactions of tapasin with MHC-I (9, 11). Efforts to produce complexes of tapasin with MHC-I molecules exploited various tricks to facilitate the interaction of the molecules that had been successful with TAPBPR (9, 11). Müller et al. took advantage of the stabilizing effect of ERp57 on tapasin and used the photolysis of bound peptide to generate multimeric complexes of human tapasin-Erp57 bound to the mouse H2-D^b^-human β_2_m heterodimer. They purified the higher order complexes and obtained X-ray quality crystals that diffracted to 2.7 Å resolution. Jiang et al. adapted an approach pioneered for MHC-II structural studies (60) and previously applied to TAPBPR in which carboxyl-terminally truncated peptides were disulfide linked to MHC-I molecules with a cysteine substitution in the α1 domain to permit refolding of molecules with partially empty peptide binding grooves. Binding studies of human tapasin to human HLA-B*44:05 revealed increased affinity for HLA-B molecules with partial occupancy of the peptide binding groove (9), and, indeed, such a complex crystallized and yielded good diffraction data to 3.1 Å. In addition, structures of human tapasin bound independently with each of two different anti-tapasin monoclonal antibodies were reported (9), and permitted modeling of the 11 to 20 loop of tapasin.

**Figure 4:**
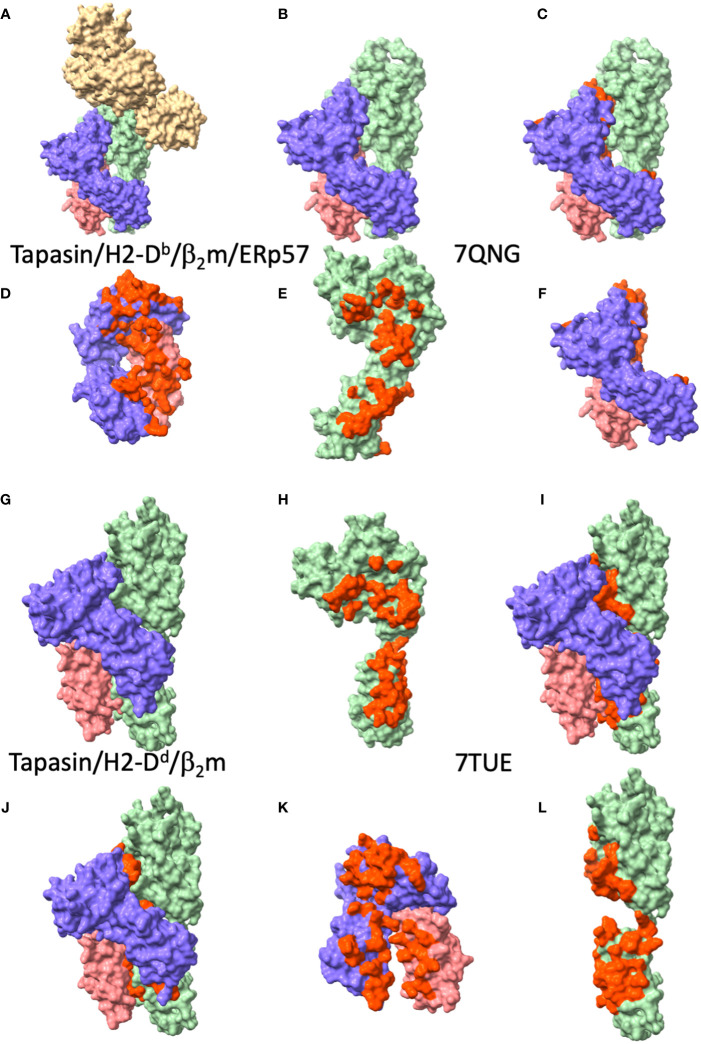
Tapasin/MHC-I interfaces reveal large area of contact to MHC-I and β_2_m. **(A-F)** views of tapasin/H2-D^b^/β_2_m/ERp57 (PDBID: 7QNG (11)), **(A)** complete complex, ERp57, tan; tapasin, light green; H2-D^b^, purple; β_2_m, coral. **(B)** tapasin/H2-D^b^/β_2_m, without tapasin (from PDBID: 7QNG (11); **(C)** tapasin/H2-D^b^/β_2_m with tapasin/MHC-I interface residues red. **(D)** H2-D^b^/β_2_m, with residues contacting tapasin, red; **(E)** tapasin, with residues contacting H2-D^b^/β_2_m, red; **(F)** H2-D^b^/β_2_m with residues contacting tapasin, red. **(G-L)** views of tapasin/HLA-B*44:05/β_2_m (PDBID: 7TUE (9)) complex, **(G)** complete complex, tapasin, light green; HLA-B*44:05, purple; β_2_m, coral. **(H)** tapasin from 7TUE with residues contacting HLA-B*44:05/β_2_m, red; **(I)** complete complex with interface residues of tapasin colored red; **(J)** complete complex with interface residues of HLA-B*44:05/β_2_m colored red; **(K)** HLA-B*44:05/β_2_m with tapasin contacting residues colored red; **(L)** tapasin with HLA-B*44:05/β_2_m contacting residues red (this is a 90° rotation from **(H)**.

The general disposition of tapasin to the MHC-I in the two structures is largely the same as that of TAPBPR (see **Figure 4**), by which the concave surface of tapasin, like the similar surface of TAPBPR, nestles the MHC-I as a baseball glove holds a ball. Superposition of the tapasin moieties from the two structures reveals an RMSD of 3.15 Å, a considerable difference, due largely to differences in the disposition of the membrane proximal IgC domains of the two structures (compare **Figure 4D** with **Figure 4K**). This kind of domain flexibility is a common feature among multi-domain proteins. Whether some of the differences may be due to the potential stabilizing influence of the ERp57 in the trimolecular (MHC-I/tapasin/ERp57) complex requires further experimental test. Both structures reveal the influence of tapasin on the general conformation of the MHC-I peptide binding groove as compared with unliganded MHC-I. Again, as in the TAPBPR structures, no peptide ligand could be visualized in either of the tapasin/MHC complexes.

The loop of tapasin residues 11 to 20 is in roughly the same position as the longer comparable TAPBPR loop 22 to 36. Because of the lack of electron density of this loop, Jiang et al. examined this region in detail in the additional crystal structures of tapasin in complex with the monoclonal antibodies PaSta1 (61) and PaSta2 (9, 18). As predicted from their behavior in immunoprecipitation experiments, the antibodies bound at either a site competitive with the general region where MHC-I binds (PaSta2) or at a site on the opposite face of the molecule so that it can immunoprecipitate the complete MHC-I complex (PaSta1). Most importantly, in the tapasin-PaSta1 complex, the region of tapasin from Trp8 to Leu26 was in excellent electron density, permitting appropriate model building, revealing the loop to be perched above the MHC α-helices and distant from the floor of the F pocket. Again, for tapasin, as for TAPBPR, little convincing evidence supports a competitive model for the effects of this loop on peptide binding or release. Additional views of the tapasin 189-195 loop (analogous to residues 209-213 of TAPBPR) confirm the flexibility of this region. Comparison of the IgC domain of tapasin when bound to MHC-I or not, or of the α3 domain of MHC-I molecules either in complex with tapasin or not supports the view that interactions among the three membrane proximal domains—MHC-I α3, β_2_m, and IgC of tapasin—contribute dynamic interactions that stabilize a peptide-free state.

In the context of these structural studies, dynamics simulations have offered an additional perspective on the mechanisms by which tapasin contributes both to the stabilization of empty MHC-I molecules and the induction of peptide release from those complexed with low affinity peptides (62, 63). Understanding particular preferences of tapasin for a range of HLA types, occasionally those that differ even by a single amino acid polymorphism (64–66) provides further incentive for additional computational simulation and structural studies.

## 3 Summary and a view of the future

Tapasin-mediated peptide loading is a key step in the normal development of the immune system and for immune surveillance for neoplastic and infected cells. Deciphering nature’s solution to how a monomorphic chaperone such as tapasin engages many representatives of a polymorphic client poses a formidable challenge. The recently reported structures of tapasin-MHC-I complexes suggest that flexibility and dynamism of both tapasin and MHC-I are part of the answer. Here, we have summarized the current understanding that TAPBPR as well as tapasin in the PLC function by interacting dynamically and influence the structure of MHC molecules globally by contacting MHC-I across a broad interface with the MHC H chain and β_2_m resulting in MHC molecules that are either free of peptide or in a peptide-receptive state. As we enter into an era where experimental structural biology yields some of its insight to the triumphs of computational prediction (67–70), it is important to maintain the conviction that experimental observation forms the basis of our understanding of protein-protein interactions. Efforts to isolate functional components either experimentally or computationally, however, are fraught with the dangers of simplification of inherently complex systems. We must continue to explore new experimental approaches and we must remain receptive to and skeptical of models that almost always are based on insufficient data. Exploration of the complexities of immune recognition is only one of many scientific undertakings that offers at least partial solutions to autoimmunity, cancers, and newly evolving infectious agents.

## Author contributions

DM, JJ, and KN outlined the original draft of this review. All authors participated in detailed discussion of the ideas and wrote different parts of the text. DM, JJ, JA, LB, and KN prepared and revised various Figures. All authors contributed to the article and approved the submitted version.
